# Empiric Azithromycin in COVID-19 Impacts the Respiratory Microbiome and Antimicrobial Resistome without Anti-inflammatory Benefit

**DOI:** 10.21203/rs.3.rs-6875205/v1

**Published:** 2025-06-20

**Authors:** Charles Langelier, Abigail Glascock, Cole Maguire, Hoang Van Phan, Emily Lydon, Carolyn Calfee, David Corry, Farrah Kheradmand, Lindsey Baden, Rafick-Pierre Sekaly, Grace McComsey, Elias Haddad, Charles Cairns, Bali Pulendran, Ana Fernandez-Sesma, Viviana Simon, Jordan Metcalf, Nelson Higuita, William Messer, Mark Davis, Kari C. Nadeau, Monica Kraft, Chris Bime, Joanna Schaenman, David Erle, Mark Atkinson, Lauren I R Ehrlich, Esther Melamed, Ruth Montgomery, Albert Shaw, Catherine Hough, Linda Geng, Annmarie Hoch, David Hafler, Alison Augustine, Patrice Becker, Bjoern Peters, Al Ozonoff, Seunghee Kim-Schulze, Florian Krammer, Steven Bosinger, Walter Eckalbar, Matthew Altman, Michael Wilson, Leying Guan, Holden Maecker, Hanno Steen, Joann Diray-Arce, Nadine Rouphael, Steven Kleinstein, Elaine Reed, Ofer Levy, Victoria Chu

**Affiliations:** University of California, San Francisco; Chan Zuckerberg Biohub; The University of Texas at Austin, Austin, TX 78712, USA; UCSF; University of California San Francisco; University of California San Francisco; IMPACC Network; Baylor College of Medicine; Baylor College of Medicine; Brigham and Women’s Hospital; Case Western Reserve University; Case Western Reserve University; Drexel University; Drexel University/Tower Health Hospital; Stanford University School of Medicine; Icahn School of Medicine at Mount Sinai; Icahn School of Medicine at Mount Sinai; University of Oklahoma Health Sciences Center; U Oklahoma; OHSU; Stanford University School of Medicine; Stanford University; University of Arizona; University of Arizona; UCLA; UCSF; University of Florida; University of Texas at Austin; The University of Texas at Austin; Yale School of Medicine; Yale University School of Medicine; Oregon Health Sciences University; Stanford University School of Medicine; Harvard; Yale University; National Institute of Allergy and Infectious Diseases/National Institutes of Health; National Institute of Allergy and Infectious Diseases/National Institutes of Health; La Jolla Institute for Allergy and Immunology; Boston Children’s Hospital; Icahn School of Medicine at Mount Sinai; Icahn School of Medicine at Mount Sinai; Emory University; University of California, San Francisco; Benaroya Research Institute; University of California, San Francisco; Yale University; Stanford University School of Medicine; Boston Children’s Hospital; Harvard; Emory University; Yale University; University of California, Los Angeles; Boston Children’s Hospital; UCSF

## Abstract

Azithromycin is often prescribed unnecessarily for respiratory infections, many of which are viral. During the COVID-19 pandemic, its use was widespread, in part due to alleged therapeutic benefits, which have since been disproven. Here, we sought to understand the impact of azithromycin exposure on the respiratory microbiome, antimicrobial resistome, and host immune response in a prospective multicenter cohort of 1164 patients hospitalized for SARS-CoV-2 infection. Using longitudinal nasal metatranscriptomics, we compared patients treated with azithromycin (n=366, 31.4%) to those who received no antibiotics (n=474, 40.7%) or antibiotics other than azithromycin (n=324, 27.8%). We found that azithromycin treatment altered the community composition of the nasal microbiome, reducing bacterial relative abundance, increasing fungal relative abundance, and increasing potentially pathogenic taxa such as *Klebsiellaand Staphylococcus*. Azithromycin treatment was most notably associated with increases in the number of detectably expressed macrolide/lincosamide/streptogramin (MLS) antimicrobial resistance genes, as well as their relative proportion in the resistome, with changes observable after one day of exposure. Of the MLS resistance genes, the expression of *ermC, msrA* and *ermX* increased the most in patients receiving azithromycin. Correlation analyses demonstrated that MLS resistance gene expression was significantly associated with the abundance of several taxa, including both commensal (e.g., *Dolosigranulum, Corynebacterium*) and potentially pathogenic genera (e.g., *Streptococcus, Staphylococcus*). Assessment of the peripheral blood and upper airway host transcriptome demonstrated no differences in the expression of inflammatory genes. Taken together, our findings demonstrate that azithromycin treatment in COVID-19 leads to dysbiosis of the upper respiratory microbiome and changes in the expression of MLS resistance genes, without apparent anti-inflammatory benefit.

## Introduction

Antimicrobial resistance (AMR) is one of the top global health threats facing humanity^1^ and is increasingly hindering the effective treatment of respiratory infections^2^. Rates of hospital-onset AMR infections dramatically increased during the SARS-CoV-2 pandemic^3^, complicating the treatment of COVID-19 and reversing the prior downward trend in deaths from drug resistant pathogens^4^. While the underlying reasons for this were multifactorial, the overuse of broad spectrum antibiotics in COVID-19 patients was a notable contributor^3,5–9^.

Azithromycin, a World Health Organization essential medicine^10^, is one of the most widely used antibiotics in human healthcare with > 40 million prescriptions annually in the United States (U.S.) alone^11^. Azithromycin overuse has been well documented in the outpatient setting^12^, where an estimated 30% of antibiotic prescriptions are inappropriate^13^. During the first year of the COVID-19 pandemic, azithromycin became one of the most commonly used antibiotics in hospitalized patients as well^9,14^. This was driven in part by early studies suggesting possible antiviral activity^15,16^, and prior work demonstrating anti-inflammatory properties of macrolide antibiotics^17,18^. Randomized clinical trials, however, subsequently demonstrated that azithromycin conferred no clinical benefit in the treatment of COVID-19^19,20^. Nonetheless, many medical centers initially incorporated azithromycin into their COVID19 treatment guidelines, and public misinformation continues to drive overprescription of the drug^21^.

Recent work has found that azithromycin exposure can alter the human microbiome and its reservoir of antimicrobial resistance genes, termed the resistome^22–24^. For instance, secondary analyses of the MORDOR (Macrolide Oraux pour Réduire les Décès avec un Oeil sur la Résistance) clinical trials found that biannual mass azithromycin distribution to African children led to an increase in the abundance of both macrolide and other AMR gene (ARGs) classes in the gut microbiome^22^. In addition, adults with asthma randomized to thrice weekly azithromycin over 12 months had an increase in PCR copy number of macrolide resistance genes in sputum samples compared to controls^25^.

Despite being the most common scenario for its use, no studies have yet assessed the impact of azithromycin on the respiratory microbiome in the context of empiric prescription for acute respiratory infection. Furthermore, no studies of azithromycin exposure have yet incorporated metatranscriptomics, which can assess both bacterial 16S rRNA abundance and ARG expression, providing a functional profile of the actively expressed resistome^26,27^.

To address these gaps, we carry out respiratory metatranscriptomics in a prospective cohort of 1164 adults hospitalized for COVID-19, and study the impacts of azithromycin exposure. We find marked changes in the respiratory microbiome, including increases in detectably expressed macrolide resistance genes and their proportional representation in the airway resistome, without evidence of antiviral or immune modulating benefit. Taken together, our findings offer new insights into the adverse effects and biological consequences of empiric azithromycin exposure during viral infection.

## Results

### Cohort

**Figure 1:**
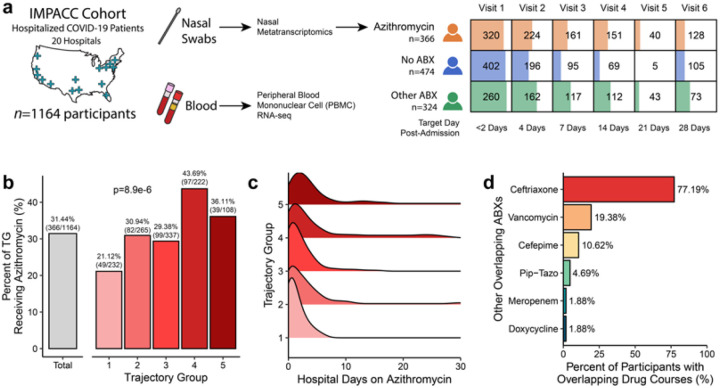
Study overview and cohort demographics. **a** Graphical overview demonstrating geographic location of IMPACC cohort study sites, sampling approach, and antimicrobial exposure groups studied including (n=366, 31.4%) treated empirically with azithromycin +/− other antibiotics, (n=474, 40.7%) who received no antibiotics, and (n=324, 27.8%) received antibiotics other than azithromycin. **b** Bar plot demonstrating percent of patients within each COVID-19 trajectory group (colors) exposed to azithromycin. Grey reference bar indicates percent of patients within the cohort treated with azithromycin. **c**Density plot highlighting distribution of azithromycin treatment with respect to days of hospitalization. **d** Bar plot depicting antimicrobials most frequently co-prescribed with azithromycin.

We carried out a prospective observational study of 1164 adults hospitalized for COVID-19 enrolled in the multicenter IMmuno Phenotyping Assessment in a COVID-19 Cohort (IMPACC)^28–30^ between May 2020 and March 2021 (Fig. 1a). Previously established COVID-19 outcome trajectory groups (TGs)^29^ were utilized to group patients based on disease severity. TGs ranged from 1 (lowest severity) to 5 (death within 28 days)^29^. Administration of azithromycin and other antibiotics was tracked following admission and throughout hospitalization. Of 1164 COVID-19 patients studied, 366 (31.4%) were treated empirically with azithromycin ± other antibiotics (Azithro group), 474 (40.7%) received no antibiotics (No-Abx group), and 324, (27.8%) received antibiotics other than azithromycin (Other-Abx group) (**Supp. Table 1 and 2**).

Empiric azithromycin administration was greatest among patients with the highest COVID-19 severity (Fig. 1b), although compared to those in the Other-Abx group, azithromycin-treated patients had less severe disease (**Supp. Table 1**). The median number of azithromycin treatment days was 2 (IQR 1–4 days, range 1–35 days), which significantly differed across TGs (p = 0.03, **Supp. Fig. 1**). Azithromycin was administered in most (98.2%) patients within 1 week of hospital admission (Fig. 1c). Patients treated with azithromycin were most likely to have been co-administered ceftriaxone (77.2%) or vancomycin (19.4%) (Fig. 1d). Sex or race did not differ based on azithromycin usage (**Supp. Table 1**).

### Impact of azithromycin exposure on the respiratory microbiome

**Figure 2:**
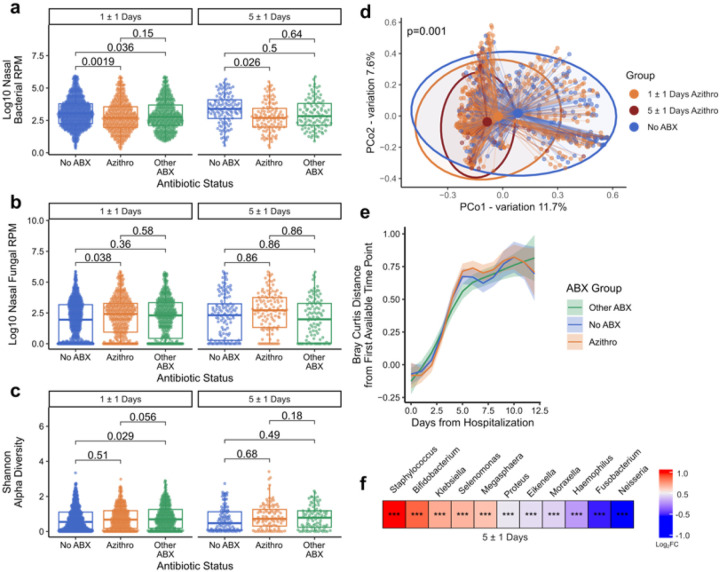
Azithromycin treatment disrupts the respiratory microbiome and mycobiome. **a** Total bacterial abundance in the nasal microbiome, measured by reads per million (RPM), comparing patients treated with azithromycin (orange), other antibiotics (green) or no antibiotics (blue) after 1 ±1 day (left) or 5 ±1 days (right) of antimicrobial treatment or hospitalization (controls). **b** Total fungal abundance in the nasal microbiome, measured by reads per million (RPM), highlighting differences between antimicrobial treatment groups. **c** Alpha diversity of the nasal microbiome highlighting differences between antimicrobial treatment groups. **d** Principal coordinate analysis of Bray-Curtis dissimilarity reveals compositional differences of the nasal microbiome based on azithromycin treatment for 1 ±1 day (orange) or 5 ±1 days (red) in comparison to no antibiotic exposure (blue). Significance calculated with PERMANOVA. **e** Bray-Curtis dissimilarity distances over time within the nasal microbiome compared to the earliest date of sampling following hospital admission. **f** Heatmap highlighting differentially abundant genera in patients treated with azithromycin for 5 ±1 days compared to those who received no antibiotics or other antibiotics. Taxa enriched with azithromycin treatment are in red, those depleted are in blue. FC = fold change. Stars correspond to statistical significance (*** = p < 0.001). For boxplots, the box limits correspond to the interquartile range (IQR) and the center line the median. The lower whisker extends to the smallest value within 1.5 * IQR below Q1, and the upper whisker extends to the largest value within 1.5 * IQR above Q3.

We first examined the impact of azithromycin on the upper respiratory tract microbiome using metatranscriptomic RNA sequencing (RNA-seq) of nasal swab (NS) samples collected at six timepoints over 28 days following hospital admission. These analyses were adjusted for age quintile, sex, severity TG, days of hospitalization, patient, receipt of corticosteroids, and receipt of the six most common antibiotics aside from azithromycin. We found that azithromycin treatment for 5 ±1 days, a common duration of prescription^31^, was associated with a significant decrease in bacterial abundance in the airway (adjusted p value (p_adj_) = 0.026), with an effect observable within 1 ±1 day (p_adj_ = 0.0019, Fig. 2a). Other antibiotics also led to a decrease in upper respiratory bacterial abundance after 1 ±1 days (p_adj_ = 0.036) but not at the later timepoint (Fig. 2a).

Assessment of the mycobiome demonstrated that receipt of azithromycin was associated with an increase in fungal relative abundance in the upper airway after 1 ±1 days (padj = 0.038, Fig. 2b), with a time-dependent increasing trend observed over 5 days of azithromycin administration (Supp. **Fig. 2b**). No differences in upper airway microbiome alpha diversity were observed based on azithromycin treatment status after 5 ±1 days, although a significant increase was seen early on (p_adj_ = 0.029, Fig. 2c). Significant differences were found in the microbiome community composition based on azithromycin expsoure, measured by Bray-Curtis dissimilarity index (PERMANOVA p = 0.001, Fig. 2d). A comparison of the trajectories for Bray-Curtis distances versus the earliest timepoint for each patient demonstrated marked shifts in community composition over time, independent of antibiotic exposure (Fig. 2e).

Differential taxonomic abundance analysis demonstrated that azithromycin exposure was associated with enrichment of potentially pathogenic taxa in the upper airway including *Staphylococcus* and *Klebsiella* species, and depletion of several typically commensal taxa such as *Neisseria* and *Fusobacterium* (Fig. 2f). We also tested whether azithromycin treatment associated with any changes in SARS-CoV-2 relative abundance in the upper airway. We observed no differences with respect to either the Other Abx or No Abx groups after 5 ±1 days of treatment (**Supp. Fig. 3**).

### Impact of azithromycin exposure on the respiratory antimicrobial resistome

**Figure 3:**
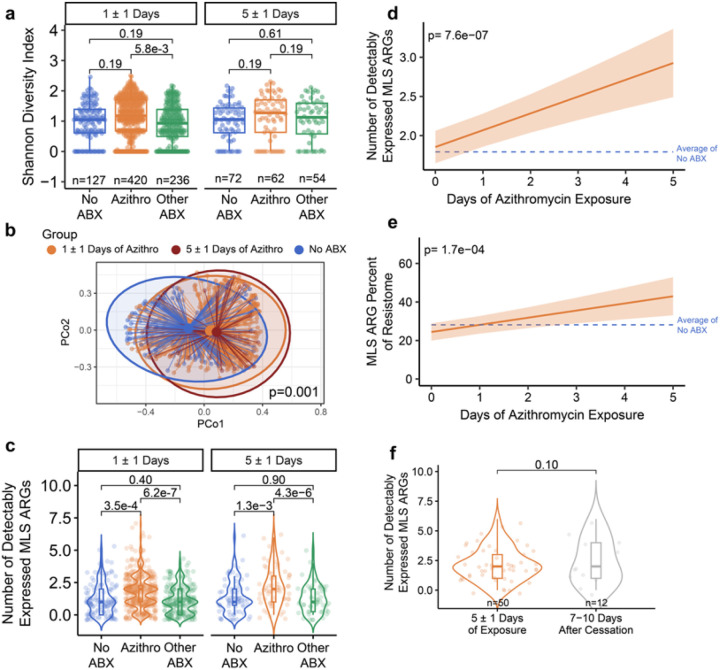
Azithromycin exposure disrupts the respiratory resistome. **a** Alpha diversity of the nasal antimicrobial resistome highlighting differences between patients treated with azithromycin (orange), other antibiotics (green) or no antibiotics (blue) at 1 ±1 day (left) or 5 ±1 days (right) of antimicrobial treatment or hospitalization (controls). **b** Compositional differences of the nasal resistome based on azithromycin treatment for 1 ±1 day (orange), azithromycin treatment for 5 ±1 days (dark orange), or no antibiotic treatment (blue). **c** Number of detectably expressed MLS resistance genes (ARGs) based on antibiotic treatment groups. **d** Generalized additive mixed model (GAMM) demonstrating changes in the number of detectably expressed MLS resistance genes over time. **e** GAMM model demonstrating longitudinal changes in the proportional representation of MLS resistance genes (orange) in the nasal resistome over time. **f** Number of detectably expressed MLS resistance genes after 5 ±1 days of exposure compared to 7 to 10 days after azithromycin cessation. For boxplots, the box limits correspond to the interquartile range (IQR) and the center line the median. The lower whisker extends to the smallest value within 1.5 * IQR below Q1, and the upper whisker extends to the largest value within 1.5 * IQR above Q3. For violin plots, the shape of the violin represents the kernel density estimate of the data, with tails trimmed to the upper and lower ranges of the data. For GAMM plots, a smoothed curve representing the estimated non-linear relationship between variables is plotted. The center line represents the predicted value, and the shaded area denotes the 95% confidence interval.

To understand the impact of azithromycin exposure on the respiratory antimicrobial resistome, we first compared Shannon Diversity Index across groups. Azithromycin exposure was associated with an increase in the alpha diversity of the upper airway resistome at the early (1 ±1 day) treatment timepoint compared with the Other-Abx group (p_adj_ = 5.8e-3) but not with the No Abx controls (Fig. 3a). A shift in the composition of the antimicrobial resistome upon azithromycin exposure was also evident, with significant differences in Bray Curtis dissimilarity indices observed at 1 ±1 day and 5 ±1 days of azithromycin treatment versus controls (PERMANOVA p = 0.001, Fig. 3b).

Since azithromycin is a macrolide class antibiotic, we next focused on ARGs conferring resistance to the macrolide, lincosamide and streptogramin (MLS) class of antibiotics. We found that azithromycin exposure was associated with a significantly greater number of detectably expressed MLS genes compared to the No-Abx or Other-Abx groups at both the early (p_adj_ = 3.5e-4 and 6.2e-7, respectively) and late (p_adj_ = 1.3e-3 and 4.3 e-6, respectively) timepoints (Fig. 3c). Longitudinal modeling subsequently demonstrated that days of azithromycin exposure resulted in a significant increase in the number of detectably expressed MLS ARGs in the upper respiratory microbiome (p_adj_ = 7.6e-7, Fig. 3d).

To comparatively evaluate the impact of azithromycin exposure across different ARG classes, we assessed longitudinal changes in the fraction of the resistome represented by each class. We found that after 5 ±1 days of azithromycin exposure, MLS ARGs increased from 24.5% to 42.9% of the resistome (p_adj_ = 1.7e-4, Fig. 3e). Notably, the enrichment in MLS ARGs, both in terms of ARG richness and as a percent of the resistome, persisted even 7–10 days after cessation of azithromycin (Fig. 3f and **Supp. Fig. 4**).

### Correlations within the resistome and microbiome

**Figure 4:**
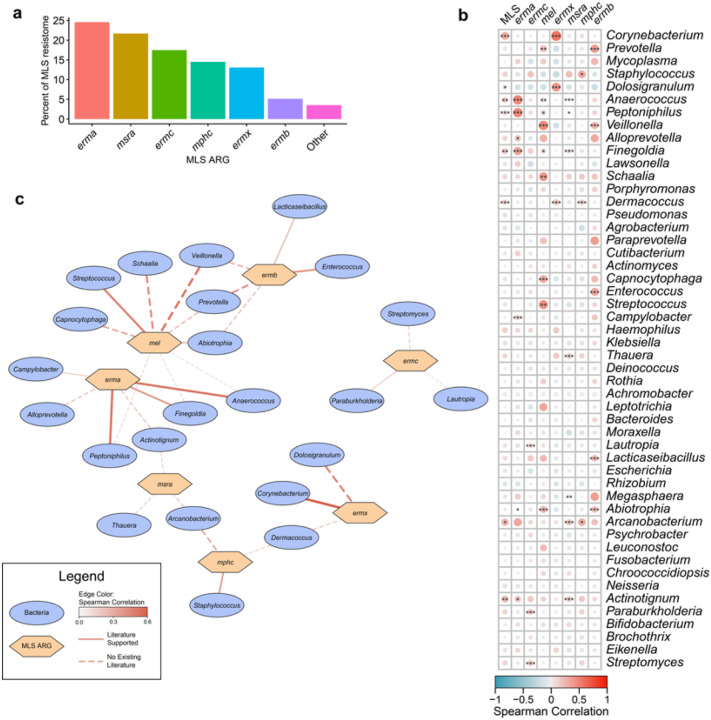
Correlations within the respiratory resistome and microbiome. **a** Percent of the MLS resistome across the full cohort comprised by individual MLS ARGs. **b** Correlations between relative abundance of MLS resistance genes (DPM) and bacterial genera (RPM). Color bar reflects Spearman correlation coefficient. ***p_adj_ < 0.001; **p_adj_ < 0.01; *p_adj_ < 0.05. **c** Network plot demonstrating significant correlations between bacterial taxa and MLS resistance genes. Taxa depicted by blue ovals. Resistance genes depicted by orange hexagons. Weight of edges and color represent spearman correlation coefficient. Solid lines show relationships supported by literature. Dashed lines show relationships where no association has previously been described in the literature.

To assess relationships between macrolide resistance genes and bacterial taxa within the upper airway microbiome, we performed multi-dimensional correlation analyses (Fig. 4b). Significant positive correlations were found between several MLS genes and both potentially pathogenic (e.g., *Staphylococcus, Streptococcus*) as well as common commensal (e.g., *Corynebacterium*^32^, *Dolosigranulum*^33^) genera (Figs. 4b, 4c).

### Impact of azithromycin on host inflammatory responses

Prior studies have found that azithromycin can confer anti-inflammatory properties, leading to off-label use of the antibiotic as an immune modulatory agent^17,18^. We thus sought to understand whether azithromycin exposure in the setting of acute COVID-19 was associated with changes in host inflammatory gene expression in the airway or blood by carrying out differential gene expression analyses. No differentially expressed genes were identified (false discovery rate < 0.05) in either anatomical compartment after 5 ±1 days of treatment (**Supp. Tables 3, 4**), suggesting that azithromycin does not meaningfully attenuate pathologic inflammatory responses in hospitalized COVID-19 patients.

## Discussion

In a large multicenter cohort of hospitalized COVID-19 patients, empiric azithromycin treatment was associated with changes in the upper respiratory tract microbiome, mycobiome and antimicrobial resistome. We observed a significant expansion of detectably expressed macrolide resistance genes after 5 ±1 days of azithromycin treatment, with effects in some cases observed within a few days. In addition, we found that azithromycin treatment was associated with changes in the composition of the upper airway microbiota including enrichment of *Klebsiella* and *Staphylococcus* species, and an increase in the burden of fungal taxa in the upper respiratory tract. Together, our findings demonstrate that inappropriate azithromycin use in patients with viral respiratory infections can drive expansion of macrolide resistance determinants and disrupt the composition of the airway microbiome.

Prior work examining mass azithromycin treatment in African children^22,24^ found a concerning relationship between exposure to this drug and an increase in macrolide resistance genes in the gut microbiome after four years. We build on these important findings by demonstrating effects in the respiratory tract and at the transcriptional level, detectable within a few days of antibiotic treatment. Importantly, we find that azithromycin leads to not only an increase in the potential for resistance within the microbiome, but a functional impact on the expression of MLS resistance genes.

Macrolide-resistant *Streptococcus pnuemoniae* and *Streptococcus pyogenes* are considered urgent threats by the U.S. Centers for Disease Control and Prevention^34^. Perhaps it is not surprising that macrolide resistance in these species has increased over the past decade given our results, and considering that 30% of antibiotics prescribed in outpatient settings have been deemed inappropriate or unnecessary^13^. Given that a large fraction of azithromycin prescriptions are written for children^12^, this is particularly concerning, as they may become colonized with macrolide-resistance bacteria at an early age due to unnecessary exposure to this drug.

Azithromycin is used prophylactically for chronic obstructive pulmonary disease^35^, cystic fibrosis/bronchiectasis^36^, lung transplantation^37^, HIV/AIDS^38^ and other conditions. While few studies have examined the impact of azithromycin prophylaxis on the respiratory resistome, a recent study of asthma patients found an increase in macrolide resistance genes using multiplex PCR in the sputum microbiome after 12 months^25^. Further work is needed to understand the impact of azithromycin prophylaxis on the upper and lower respiratory microbiome and resistome in these patient populations.

A sub-analysis of the MORDOR trial found that four years of biannual azithromycin treatment in African children reduced mortality but led to an increase in macrolide resistant *Streptococcus pneumoniae* cultured from the nasopharynx^24^. Consistent with these prior microbiological observations, our correlation analysis suggested that both *Streptococcus* and *Staphylococcus* species, encompassing some of the most important bacterial pneumonia pathogens, may harbor these resistance determinants. In addition, we found relationships between MLS resistance gene expression and the abundance of commensal and contextually pathogenic taxa, such as *Corynebacterium* species.

In the MORDOR trial, mass azithromycin treatment was also found to cause an increase in the burden of non-macrolide resistance genes in the gut microbiome^22^. In contrast, we found relatively few off-target effects on other classes of ARGs in the upper airway. One possible explanation may lie in the age and demographic differences of the studied populations. For instance, the burden of ARGs in both the gut^39,40^ and respiratory microbiome increases with age, potentially due to lifetime antibiotic exposures^26^. It is possible that our cohort of hospitalized adults in the U.S. may have had a higher baseline burden of ARGs compared to African children living in rural settings. Alternatively, differences may be attributable to sampling of the respiratory versus gut microbiome, or the use of metatranscriptomics versus metagenomic DNA sequencing.

Prior work has demonstrated that azithromycin has immune-modulating potential^17,18^, findings that have encouraged its use in patients with cystic fibrosis, chronic obstructive pulmonary disease^41^, and other inflammatory diseases. Azithromycin use early during the COVID-19 pandemic was in part driven by the idea that it might attenuate harmful inflammatory responses, as well as a now-retracted publication purporting clinical benefit when combined with hydroxychloroquine^42^. Randomized clinical trials, however, subsequently found no therapeutic benefit of azithromycin for COVID-19^19,20^. Consistent with this, we observed no significant associations between azithromycin treatment and inflammatory gene expression, or viral load, in either the airway or blood of hospitalized COVID-19 patients.

Strengths of our study include a large multicenter cohort, detailed clinical phenotyping, use of respiratory metatranscriptomics, and rigorous quality control of clinical and biological data. As with any research, our study also has limitations. These include the observational study design and the use of short read sequencing, which precluded definitively linking macrolide resistance genes to specific taxa. In addition, our analyses were limited to the upper airway and thus may not reflect microbial changes occurring in the lungs.

Antibiotic administration data were extracted manually by clinical research coordinators at each study site, an approach susceptible to human error. Azithromycin treatment, however, was confirmed by an independent adjudicator for every patient. Future randomized clinical trials - ideally that include airway and gut microbiome sampling as well as bacterial culture - are needed to more fully characterize the impact of exposure to azithromycin and other antibiotics on the human microbiome and resistome.

In sum, we find that azithromycin exposure in hospitalized COVID-19 patients is associated with compositional changes in the airway microbiome and expansion of macrolide resistance genes. Taken together our findings suggest that empiric use of azithromycin in patients with viral respiratory infections may lead to adverse impacts and contribute to antimicrobial resistance.

## Methods

### Study Design, Clinical Cohort, Inclusion and Ethics

IMPACC is a prospective longitudinal study that enrolled 1164 hospitalized COVID-19 patients, as previously described in detail^28–30,43,44^. Participants 18 years and older were recruited from 20 hospitals across 15 academic biomedical centers within the United States and confirmed to be SARS-CoV-2 positive by reverse transcription PCR (RT-PCR) testing. No participants were vaccinated for SARS-CoV-2 at time of enrollment nor during their hospitalization. To better categorize patients into different COVID19 severity groups, they were classified into one of five trajectory groups using latent class mixed modeling of the degree of respiratory illness and external oxygen administration^29^.

The Department of Health and Human Services Office for Human Research Protections (OHRP) and NIAID concurred that the IMPACC study qualified for public health surveillance exemption. The study protocol was reviewed by each site’s institutional review board (IRB), with twelve sites conducting as a public health surveillance study, and three sites integrating the IMPACC study into IRB-approved protocols (The University of Texas at Austin, IRB 2020-04-0117; University of California San Francisco, IRB 20–30497; Case Western Reserve University, IRB STUDY20200573) with participants providing informed consent. Participants enrolled at sites operating as a public health surveillance study were provided information sheets describing the study including the samples to be collected and plans for analysis and data de-identification. Participants who requested not to participate after review of the study plan and information were not enrolled. Participants were not compensated while hospitalized but were subsequently compensated for outpatient visits and surveys. This study was registered at clinicaltrials.gov (NCT0438777) and followed the Strengthening the Reporting of Observational Studies in Epidemiology (STROBE) guidelines.

### Metatranscriptomic Sequencing

Mid-turbinate nasal swabs were collected withing 72 hours of hospital admission and at subsequent visits with target dates of 4, 7, 14, 21, and 28 days post hospital admission. The nasal swabs were stored in 1 mL of Zymo-DNA/RNA shield reagent (Zymo Research), before RNA was extracted twice in parallel from 250 uL of sample. The RNA was then purified with the KingFisher Flex sample purification system (ThermoFisher) and the quick DNA-RNA MagBead kit (Zymo Research). The duplicated RNA was pooled and aliquoted at 20 μL for the downstream RNA-sequencing. The isolated RNA was subsequently sequenced on a NovaSeq 6000 (Illumina) at 100 bp paired-end read length. The data was aligned using STAR (v2.4.3) against the GRCh38 reference genome and host gene counts were quantified using HTSeq-count (v0.4.1).

### Microbiome and Resistome Profiling

Metatranscriptomic data was processed using the open-source CZ ID pipeline (https://czid.org/)^45^. Microbiome profiling was performed within CZ ID using the Illumina mNGS pipeline (v7.1). CZ ID first removes host reads by aligning to the GRCh38 human reference genome using STAR^46^. Adapters are removed using Trimmomatic^47^ and reads are filtered for low quality using PriceSeq^48^. Duplicates are identified using czid-dedup. Low complexity reads are filtered out using the Lempel-Ziv-Welch algorithm and any remaining host reads are removed by alignment to GRCh38 using Bowtie2^49^. After performing the filtering steps, de-duplicated reads are subsampled to 2 million total reads. Taxonomic classification of remaining reads is performed on reads and assembled contigs (SPAdes)^50^ by aligning to the NCBI nucleotide (NT) and protein (NR) databases. Resistome profiles were generated using the CZ ID AMR pipeline (v0.2), which leverages the Resistance Gene Identifier (RGI) tool and the Comprehensive Antibiotic Resistance Database (CARD)^51^. Following microbiome and resistome profiling with CZ ID, background and batch correction were performed to remove contaminants and adjust for batch effects (see below). To limit the contribution of spurious hits to downstream analyses, additional filtering of microbial taxa and ARGs was performed on a per sample basis. Microbial taxa were excluded if they did not meet all of the following criteria: (1) ten hits to the NT database, (2) one hit to the NR database, (3) a minimum alignment length of 50 bases. ARGs were excluded if they did not have ≥5% read coverage breadth and meet one of the following two criteria: (1) present in ≥5% of all samples with detectable ARGs (2) depth per million (DPM) ≥1 and ≥10 hits.

### Background & Batch Correction

Negative controls (double distilled water) were processed and sequenced alongside samples in the IMPACC cohort to enable the characterization and subtraction of background contamination. The sequencing data generated from these samples was analyzed using the CZ ID metagenomic and AMR pipelines as described above. Background and batch correction was performed on the microbiome and resistome datasets separately. A negative binomial model was used to model the distribution of reads of microbial taxa/ARGs in the negative controls. Mean and dispersion parameters were then fitted to these data. Mean estimates were generated for each batch:taxon or batch:ARG pair in the negative controls, where batch corresponds to the phase of the IMPACC study (1, 2, 3A or 3B). The MASS package (v7.3.58.1) in R was used to generate a single dispersion parameter across all taxa/ARGs. P-values were adjusted for multiple testing using the Benjamini-Hochberg False Discovery Rate algorithm.

Microbial taxa/ARGs that were present at a significantly higher abundance in participant samples than in negative controls (FDR <0.1) were retained for downstream analyses.

### Single Time Point Analyses

Participants were assigned to one of three groups: those who received Azithromycin (Azithro) +/− other antibiotics, those who took only non-Azithromycin antibiotics (Other-Abx), or participants who did not receive antibiotics (No-Abx). Participants with only partially captured antibiotic start and stop dates were excluded from these analyses. The 2 time points studied for these groups were samples collected with 1 ±1 day of exposure and 5 ±1 days of exposure, applicable to the Azithromycin and Other-Abx groups. The second time point (5 ±1 days) was chosen based on the average course of azithromycin, approximately 5 days. In an attempt to match the No-Abx samples to the Azithromycin and Other-Abx samples at each time point, the distribution of days of hospitalization across those groups was examined. Based on the findings, we selected samples for the No-Abx group as follows: In the 1 ±1 day group, the samples had no antibiotic exposure and up to 40 days of hospitalization. In the 5 ±1 days group, the samples had no antibiotic exposure, and between 3–40 days of hospitalization. Samples that qualified for both time points were split evenly between time points. The number of “No-Abx” samples was also rarified (50%) to make the numbers in each group more comparable. In addition to this sample matching process for the No-Abx group, days from hospital admission was included as a covariate in statistical models to further control for slight differences. A linear mixed-effects model (using the lme4 package) was used to calculate differences between the groups at both timepoints while using age quintile, sex, severity TG, days from hospitalization, and receipt of steroids as fixed effects in the model, in combination with the participant’s enrollment site included as a random effect. For only the Azithromycin group, the number of days of the six most common co-administered antibiotics (vancomycin, ceftriaxone, cefepime, piperacillin-tazobactam, doxycycline and meropenem) were also included as fixed effects. Resulting p values were adjusted using the Benjamini-Hochberg False Discovery Rate algorithm via the p_adjust() function of the stats (v4.2.3) package.

### Generalized Additive Mixed Model (GAMM) Analyses

Various metrics were modeled over days of azithromycin exposure using the nlme (v3.1–162), lme4 (v1.1–35.5), lmerTest (v3.1–3) and ggeffects (v1.7.2) packages in R. Samples were limited to the first 10 days of hospitalization and the first 5 days of antibiotic exposure due to few patients with longer antibiotic courses. The GAMM models included fixed effects of severity TG, sex, age quintile of the participant, and whether they ever received steroids, in addition to a smoothed term for days of azithromycin usage and days from hospitalization, and participant as a mixed effect. Additionally, days of administration for the six most prevalent antibiotics in the cohort aside from azithromycin (vancomycin, ceftriaxone, cefepime, piperacillin-tazobactam, doxycycline, and meropenem) were also added to the models as smooth terms.

R model formula:

~s(Azithromycin,bs=‘cr’,k=4)+s(Ceftriaxone,bs=‘cr’,k=4)+s(Vancomycin,bs=‘cr’,k=4)+s(Cefepime,bs=‘cr’,k=4)+s(Meropenem,bs=‘cr’,k=4)+s(Piperacillin_Tazobactam,bs=‘cr’,k=4)+s(Doxycycline,bs=‘cr’,k=4)+s(event_date,bs=‘cr’)+trajectory_group+sex+discretized_admit_age_quantile+ever_steroids

### Microbiome Diversity Metrics

Alpha diversity (Shannon Diversity Index) was calculated using the diversity() function in the R package vegan(2.6–6.1). Beta diversity (Bray-Curtis dissimilarity) analysis was performed using the vegan functions vegdist(), betadisper(), permutest() and adonis2(). The beta diversity analysis was adjusted for age quintile, sex, days since hospitalization, severity TG, patient, and receipt of corticosteroids in the adonis2() function. For the resistome analysis, receipt of the six most common antibiotics aside from azithromycin (vancomycin, ceftriaxone, cefepime, piperacillin-tazobactam, doxycycline and meropenem) was also included in the model. Principal Component Analysis (PCoA) of the resistome and bacterial microbiome was also performed using cmdscale() function of the stats(v4.2.3) package.

### Differential Abundance Analysis

Bacterial microbiome profile data was converted into a phyloseq object using R packages ape (v5.8.1), ade4 (v1.7.22), and phyloseq (v1.34.0). Differential abundance analysis was performed using the R package ANCOMBC (v1.0.5) with an alpha level of 0.05 and prevalence filter (zero_cut argument) of 0.97. The analysis was adjusted for the following covariates: age quintile, sex, days from hospitalization, severity TG, patient, receipt of corticosteroids and receipt of the six most prevalent antibioticsaside from azithromycin (vancomycin, ceftriaxone, cefepime, piperacillin-tazobactam, doxycycline, and meropenem). P-values were adjusted for multiple testing using the Benjamini-Hochberg correction.

### Correlation Analyses

The 50 most abundant bacterial taxa by reads per million (RPM) were included in the correlation analysis. RPM was used as an abundance metric in lieu of raw reads to adjust for variations in sequencing depth between samples. The abundance metric used for ARGs was depth per million (DPM), which adjusts for both variations in sequencing depth between samples and variations in gene length between ARGs. Spearman correlation was performed between bacterial taxa RPM and ARG DPM using the cor() function in the stats (v4.2.3) package and the cor_pmat() function in the rstatix (v0.7.2) package in R. Results were visualized using the corrplot (v0.95) package.

### PBMC and Nasal Swab Host Transcriptional Profiling

RNA-seq and alignment against the host transcriptome was performed as previously described,^52,53^ and the deidentified, quality-controlled raw gene count files and metadata were obtained from the IMPACC study. We filtered for host protein-coding genes that had at least 10 counts in at least 20% of the samples. To identify genes that were associated with azithromycin exposure compared to No-Abx patients, we utilized the limma (v3.58.1) package in R. We modeled gene expression as a function of azithromycin exposure, corrected for age, sex, trajectory group, days since hospitalization, and exposure to corticosteroids. Then, we applied voom and duplicateCorrelation for two iterations to calculate the correlation between multiple samples from the same patients. Next, we applied the lmFit and eBayes functions to estimate the effects of Azithromycin exposure on gene expression and their P-values. Finally, the P-values were adjusted with Benjamini-Hochberg correction.

### Statistics

All code was written in R v4.2.3 or R 4.0.3. Data processing was performed using R packages dplyr (v1.1.4) and tidyverse (v2.0.0). Plots were generated using R packages ggplot2 (v3.5.1), ggpubr (v0.6.0), scales (1.3.0) and ggbeeswarm (v0.7.2). Package versions for each analysis are reported in the code repository.

## Supplementary Files

This is a list of supplementary files associated with this preprint. Click to download.

- SupplementalTable3NS.csv
- SupplementalTable4PBMC.csv
- nreditorialpolicychecklistNMEDA142753.pdf
- SupplementaryMaterials.docx

## Data Availability

Data used in this study is available at ImmPort Shared Data under the accession number SDY1760 and in the NLM’s Database of Genotypes and Phenotypes (dbGaP) under the accession number phs002686.v2.p2. Source data for each figure are provided with this manuscript in the Source Data file. All code is deposited in the following Bitbucket repository: https://bitbucket.org/kleinstein/impacc-public-code/src/master/azithromycin_manuscript/.
